# Identification of 1,3,6,8-Tetrahydroxynaphthalene Synthase (ThnA) from *Nocardia* sp. CS682

**DOI:** 10.4014/jmb.2303.03008

**Published:** 2023-05-05

**Authors:** Purna Bahadur Poudel, Rubin Thapa Magar, Adzemye Fovennso Bridget, Jae Kyung Sohng

**Affiliations:** 1 Institute of Biomolecule Reconstruction (iBR), Department of Life Science and Biochemical Engineering, Sun Moon University, Asan 31460, Republic of Korea; 2 Department of Pharmaceutical Engineering and Biotechnology, Sun Moon University, Asan 31460, Republic of Korea

**Keywords:** *Nocardia*, Type III PKS, heterologous expression, Streptomyces lividans

## Abstract

Type III polyketide synthase (PKS) found in bacteria is known as 1,3,6,8-tetrahydroxynaphthalene synthase (THNS). Microbial type III PKSs synthesize various compounds that possess crucial biological functions and significant pharmaceutical activities. Based on our sequence analysis, we have identified a putative type III polyketide synthase from *Nocardia* sp. CS682 was named as ThnA. The role of ThnA, in *Nocardia* sp. CS682 during the biosynthesis of 1,3,6,8 tetrahydroxynaphthalene (THN), which is the key intermediate of 1-(*α*-L-(2-*O*-methyl)-6-deoxymannopyranosyloxy)-3,6,8-trimethoxynaphthalene (IBR-3) was characterized. ThnA utilized five molecules of malonyl-CoA as a starter substrate to generate the polyketide 1,3,6,8-tetrahydroxynaphthalene, which could spontaneously be oxidized to the red flaviolin compound 2,5,7-trihydroxy-1,4-naphthoquinone. The amino acid sequence alignment of ThnA revealed similarities with a previously identified type III PKS and identified Cys^138^, Phe^188^, His^270^, and Asn^303^ as four highly conserved active site amino acid residues, as found in other known polyketide synthases. In this study, we report the heterologous expression of the type III polyketide synthase *thnA* in *S. lividan* TK24 and the identification of THN production in a mutant strain. We also compared the transcription level of *thnA* in *S. lividan* TK24 and *S. lividan* pIBR25-*thnA* and found that *thnA* was only transcribed in the mutant.

## Introduction

**Fig. 1:**
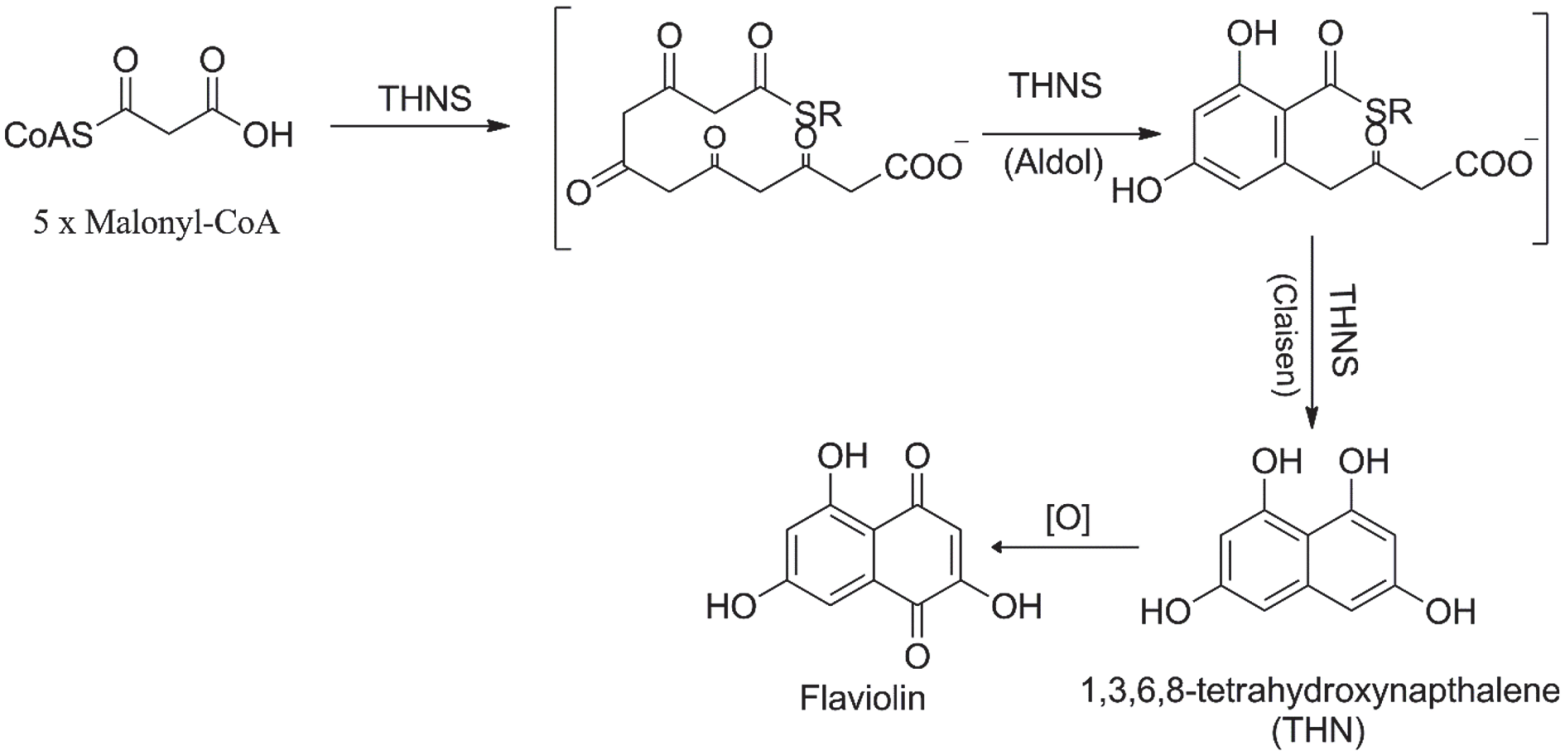
Reaction scheme of 1,3,6,8-tetrahydroxynaphthalene synthase (THNS). R = coenzyme A (CoA) or the active enzyme site, a cysteine thiol group.

Polyketide synthases (PKSs) are a group of enzymes that are responsible for the synthesis of complex and biologically active metabolites in all living organisms, ranging from microorganisms to plants. These enzymes work in a coordinated and sequential manner to produce these essential compounds [1-3]. Polyketides are a diverse family of natural products that display a vast array of biological activities, including antimicrobial, antiparasitic, antifungal, and anticancer properties. They have also various commercial applications as food additives, nutraceuticals, and pigments [4-10]. The synthesis of most polyketides involves the use of three main classes of PKSs, namely type I PKS, type II PKS, and type III PKS. These three types of PKSs use a similar mechanism of sequential decarboxylative condensations, which can take place with a diverse range of acyl-coenzyme A (CoA) substrates [11, 12-14]. Type I PKSs are mainly composed of multifunctional proteins that consist of various modules. These modules have non-iterative functions that are responsible for catalyzing one cycle of polyketide chain elongation [15]. Type II PKSs are characterized as multienzyme complexes, where each catalytic domain is encoded by a separate gene [13]. Type III PKSs are generally homodimeric enzymes with a single active site iteratively acting as condensing enzymes [1]. Type III PKSs, which are relatively small homodimeric proteins consisting of monomers weighing between 40-47 kDa, play a crucial role in the biosynthesis of aromatic polyketides in both bacterial and plant PKSs [7]. Type III PKSs are widely distributed in bacteria, plants, and fungi. The synthesis of 1,3,6,8-tetrahydroxynaphthalene (THN) occurs through the catalytic action of RppA, which utilizes five malonyl-CoA molecules to produce THN which subsequently undergoes spontaneous oxidation to form flaviolin (Fig. 1). It was the first functionally characterized bacterial THN synthase from *Streptomyces griseus* [16].

**Fig. 2:**
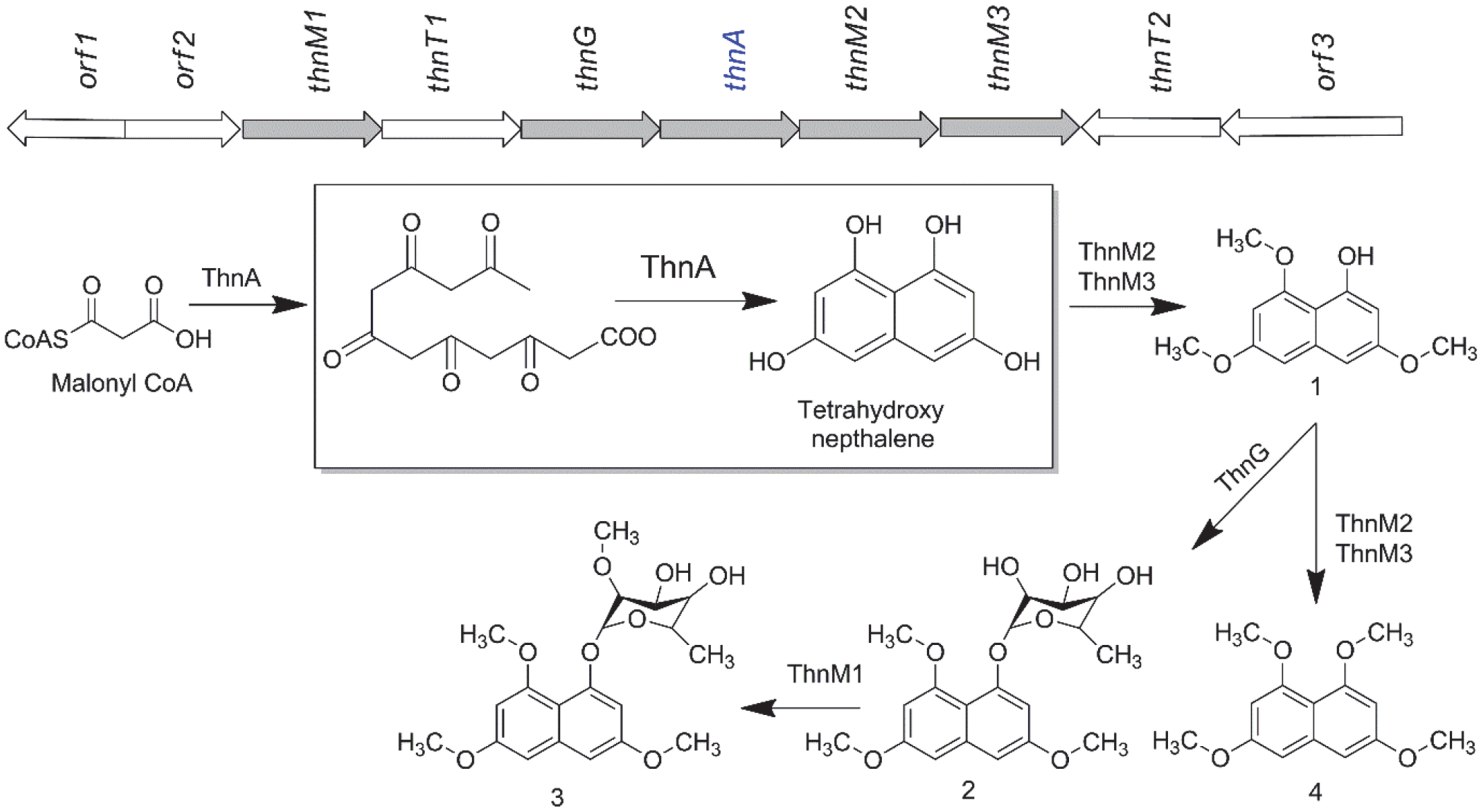
The putative biosynthetic gene cluster and proposed biosynthetic pathway of compound 3. Compound **1**: 3,6,8-trimethoxy naphthalen-1-ol; **2**: 1-(*α*-L-6-deoxy-mannopyranosyloxy)-3,6,8-trimethoxy naphthalene; **3**: 1-(*α*-L-(2-O-methyl)-6-deoxymanno-pyranosyloxy)-3,6,8-trimethoxy naphthalene, and **4**: 1,3,6,8-tetramethoxy naphthalene.

Type III PKSs are known to produce THNs as the predominant metabolites in several actinomycetes, including *S. griseus* [17], *Saccharopolyspora erythrea* [18], *S. peucetius* [6], *S. toxytricini* [19], and *Sorangium cellulosum* [20]. *Nocardia* sp. CS682 contains ThnA, one of the type III PKSs found in the *Nocardia* sp. CS682 genome [21]. Although its biological function is currently unknown, ThnA has been implicated in the biosynthesis of 1-(*α*-L-(2-*O*-methyl)-6-deoxymannopyranosyloxy)-3,6,8-trimethoxynaphthalene (IBR-3) [22]. *Nocardia* sp. produces a UV-protective compound known as IBR-3, which is formed by removing the complete PKS region of nargenicin [15]. Different enzymes like methyltransferases and glycosyltransferase are involved in the post-modification of 1,3,6,8-tetrahydroxynaphthalene (THN) during the formation of IBR-3 (Fig. 2) [15, 21, 22]. Due to the crucial role played by ThnA in forming the core THN scaffold, we carried out a sequence alignment of ThnA with established type III PKSs. This alignment yielded a high level of bootstrap support, thereby strengthening the case for heterologous expression.

In this study, we characterized the function of *thnA* by heterologous expression in *S. lividan* sp. TK24. ThnA is a key enzyme in the production of 1-(*α*-L-(2-*O*-methyl)-6-deoxymannopyranosyloxy)-3,6,8-trimethoxynaphthalene from five malonyl-CoA molecules to form THN, which undergoes further modifications by methyltransferases and glycosyltransferase enzymes involved in this pathway to yield the final product IBR-3.

## Materials and Methods

### Bacterial Strains, Plasmids, and Culture Conditions

*S. lividan* TK24 was utilized as a host for heterologous expression and cultured in 50 ml of R2YE medium, which is composed of 0.02% potassium sulfate, 10.3% sucrose, 1% magnesium chloride, 0.5% yeast extract, 0.01%disfco casamino acid, and 1% glucose. To extract genomic DNA, *Nocardia* sp. CS682 was cultured for 5 days at 37°C and 200 rpm in brain heart infusion (BHI) medium [23]. The bacterial strains *E. coli* XL1 and *E. coli* ET12567 were cultured at 37°C on either solid or liquid Luria Bertani (LB) medium. The cloning vector utilized in this study was pGEM-T Easy (Promega, USA). Recombinant DNA was constructed with the plasmid vector pIBR25 (Fig. S1A), which was placed under the regulation of an ermE promoter to facilitate the expression of DNA in Streptomyces [24]. In order to transform the recombinant DNA into the protoplasts of *S. lividan* TK24, the standard protocol utilized in our previous study was followed [25]. Standard commercial sources were utilized to purchase chemicals and reagents for biochemical and molecular biology analyses.

### Construction of Recombinants and Transformation into *Streptomyces lividans* TK24

The TIANamp bacterial DNA kit was used to isolate and purify genomic DNA from *Nocardia* sp. CS682 [21]. Polymerase chain reaction (PCR) was used to amplify the *thnA* (1,125-bp) from *Nocardia* sp. CS682 genomic DNA using the following primer pair: 5¢-GGATCCATGGCAATCTTGTGTCGAC-3¢ (forward, BamHI) and 5¢-GAATTCTCATAGGACTACCTCCCCT-3¢ (reverse EcoRI) with high fidelity *pfu* DNA polymerase (Takara, Japan). PCR analysis was performed on a thermal cycler from Takara, Japan, using the following conditions: a 7 min initial denaturation at 95°C, 30 cycles of denaturation at 92°C for 1 min, annealing at 58°C for 1 min, and extension at 72°C for 1 min. The final step involved a 7 min extension at 72°C. To prepare the PCR products for further analysis, they were purified from a 0.7% agarose gel, and then ligated into the pGEM^®^-T Easy vector from Promega, Wisconsin, USA. The BamHI-EcoRI fragment containing *thnA* was ligated into pIBR25 to construct pIBR25-*thnA* (Fig. S1). pIBR25-*thnA* was transferred into *E. coli* XL1-Blue and the plasmids were isolated and transformed into the final *E. coli* ET-12567 construct to obtain demethylated plasmid for transformation in *S. lividan* TK24 by protoplast transformation.

### Protoplast Preparation, Transformation in *S. lividan*p TK24

*S. lividan* TK24 protoplast preparation was performed as described previously [25]. Briefly, 150 μl of protoplasts were treated for 12 min with 15 μl of 0.1 mM aurintricarboxylic acid (ATA) (Sigma-Aldrich, USA) and mixed with 20 μl of plasmid DNA for 2 min. After that, a 40% (w/v) polyethylene glycol 1000 (PEG) solution from Merck-Schuchardt, Germany, was added to the mixture (200 μl) and gently mixed. The solution was then briefly centrifuged to remove PEG and the resulting sample was resuspended in protoplast buffer [26]. The transformed protoplasts were spread onto R2YE regeneration plates at 28°C for 16 h. Then, an overlay of 0.3% agar solution containing 15 μg/ml thiostrepton was added, and the plates were screened on R2YE plates with the same concentration of thiostrepton (15 μg/ml). A parallel transformation and screening were also performed using pIBR25 to generate *S. lividan* pIBR25.

### Extraction, Isolation, and In Vivo Analysis

*S. lividan* TK24 and other mutants (*S. lividan* pIBR25-thaA, *S. lividan* pIBR25) were cultivated in 50 ml of R2YE media supplemented with 15 μg/ml of thiostrepton at a temperature of 28°C for a duration of 5 days and extracted with a double volume of ethyl acetate. The organic layer was then concentrated using a rotary evaporator and the resulting concentrated sample was dissolved in a suitable amount of methanol, filtered, and subsequently examined through reverse-phase high-performance liquid chromatography (HPLC, Mightysil RP-18 GP 250-4.6, Japan), and mass analysis was performed by liquid chromatography-electron spray ionization/mass spectrometry (LC-ESI/MS) in the positive ion mode using an Acquity column (UPLC; Waters Corp., USA) coupled with a SYNAPT G2-S column (Waters Corp.). The sample was eluted using a solvent mobile phase gradient of 0.1%trifluoroacetic acid (TFA) in water and 100% acetonitrile (ACN) over a period of 0 to 12 min at a temperature of 35°C. The volume of the injected sample was 10 microliters.

### RNA Sample Preparation and Reverse Transcription PCR Analysis

To extract total RNA, each 5 ml aliquot of culture that was grown for approximately 72 h was suspended in RNA protect Bacteria Reagent (Qiagen, Germany) for a duration of 5 min. RNA isolation was carried out using the RNeasy Mini kit (Qiagen) in accordance with the guidelines provided by the manufacturer. DNase (Qiagen) was used to treat contaminating DNA in the RNA samples, and the lack of contamination was verified by PCR analysis using the RNA as a template. To assess the purity and concentration of the total RNA, a spectrophotometer (Shimadzu, UV-1601 PC) was utilized to measure the optical density at 260/280 nm. Reverse transcription PCR (RT-PCR) was performed with a QuantiTech SYBR Green RT-PCR kit (Qiagen). The primers used for *thnA* (158 bp) were 5'-CCGAGCGCTAGGAAACG-3' (forward) and 5'-ATCCGAGCTGCGCGATA-3' (reverse), and for the control housekeeping gene [*rpoB* (181 bp)] they were 5'-AGTTCGGCGAGTACGAGTC-3' (forward) and 5'-ACCTTGGCGAGGTCGTAG-3' (reverse). For the RT-PCR analysis, identical amounts of RNA from each sample were utilized. The reaction conditions involved initial cDNA synthesis at 50°C for a duration of 30 min, followed by initial denaturation at 98°C for 10 min and 45 cycles. Each cycle consisted of denaturation at 98°C for 30 sec, annealing at 55−68°C for 30 sec, and elongation at 72°C for 1 min. The amplified DNA samples were further subjected to 1–2% agarose gel electrophoresis to verify the amplification product. To verify that the amplified products were not originating from chromosomal DNA that might have contaminated the RNA preparations, negative controls were conducted using Taq DNA polymerase without reverse transcriptase. To determine whether *thnA* was expressed in *S. lividan* TK24, we cultured the wild-type, *i.e.*, *S. lividan* TK24, and *thnA* overexpressed in *S. lividan* pIBR25-*thnA* in HT medium. Samples were taken on the third day, and mRNA was isolated using a standard protocol. *RpoB* is a housekeeping gene in *S. lividan* TK24 and was used as a control.

## Results and Discussion

### Sequence and Phylogenetic Analysis of ThnA

**Fig. 3:**
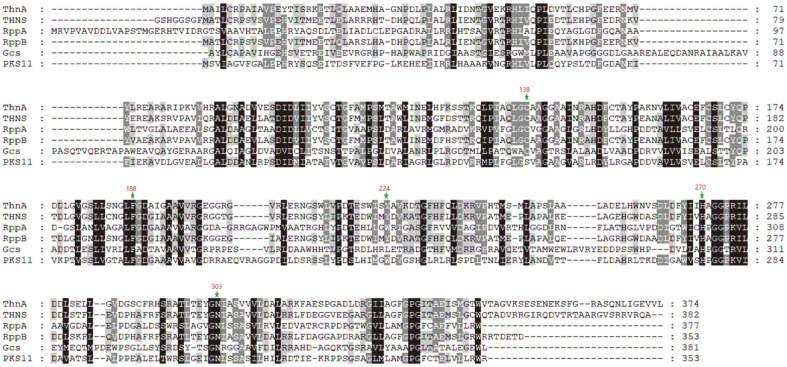
Sequence alignment of ThnA protein with other known type III PKSs. The comparison was carried out with THNS from *S. coelicolor* A3 (1U0M), RppA from *S. peucetius* (ABY71276), RppB from *S. antibioticus* (BAB91444), Gcs from *S. coelicolor* (3v7i) and PKS11 from *Mycobacterium tuberculosis* (4JAT). Catalytic motifs are marked by green stars.

The genomic analysis of *Nocardia* sp. CS682 [21] showed that the biosynthetic gene cluster (BGC) responsible for the synthesis of 1-(*α*-L-(2-*O*-methyl)-6-deoxymannopyranosyloxy)-3,6,8-trimethoxynaphthalene consisted of PKS (ThnA) and three *O*-methyltransferases (ThnM1, ThnM2, and ThnM3) (Fig. 2). ThnA was putatively annotated for the production of 1,3,6,8-tetrahydroxynaphtalene (THN) from malonyl-CoA. The *thnA* gene encodes a protein consisting of 374 amino acids (AA), and the genés overall G+C content is 56%. According to BLAST searches, *thnA* shows significant similarity with other type III PKS genes in the database. The amino acid sequence alignment of ThnA, as illustrated in Fig. 3, exhibited the existence of four extensively conserved residues, namely Cys^138^, Phe^188^, His^270^, and Asn^303^, which are present in other known PKSs. From the structure and functional studies of chalcone synthase and other PKSs, Cys^138^ serves as a nucleophile that starts the reaction and makes the enzyme-bound polyketide intermediate [7, 27, 28]. Phe^188^ provides a hydrophobic environment that promotes the decarboxylation of malonyl-CoA [29], ionic interaction of His^270^ plays a crucial role in stabilizing the thiolate anion of Cys168 [30], and Asn^303^ is responsible for catalyzing the decarboxylation of malonyl-CoA [1, 30]. Additionally, it has been previously demonstrated that Tyr^224^ assists in substrate specificity, and its aromatic ring is considered essential for ThnA to recognize malonyl-CoA as a starter unit, much like the two other aromatic amino acids, Trp and Phe [29]. The amino acid alignment and conserved domains in ThnA suggest that it could serve as a THNS. A phylogenetic tree was constructed using the protein sequences of ThnA and their closest homologs (Fig. S2). Phylogenetic tree analysis of ThnA revealed that it had the closest relationships with functionally characterized PKS.

### Heterologous Expression of *thnA*

**Fig. 4:**
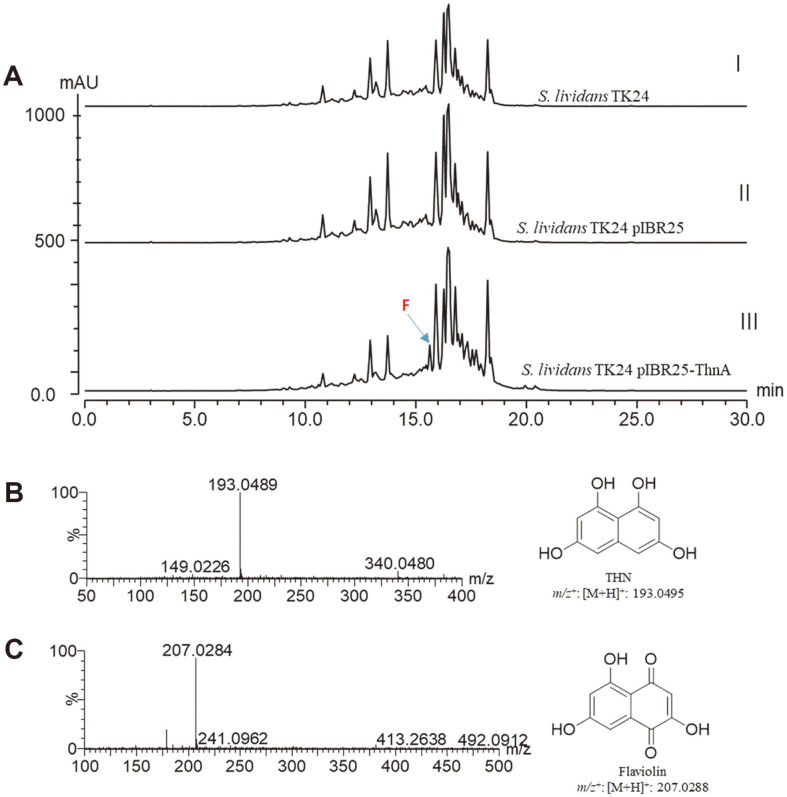
HPLC and LC-ESI/MS analysis of *in vivo* products. (**A**) HPLC patterns of compounds I) from *S. lividan* TK2, II) *S. lividan* TK24 pIBR25, *and* III) *S. lividan* TK24 pIBR25-*thnA*. (**B**) LC-ESI/MS data of THN and (**C**) LC-ESI/MS data from flaviolin from *S. lividan* TK24 pIBR25-*thnA*.

In this study, *S. lividan* TK24 was utilized as a host for heterologous expression. The construction of pIBR25-*thnA* involved cloning the DNA fragment containing *thnA* into pIBR25, which was subsequently transformed into *S. lividan* TK24 via protoplast transformation using a standard protocol [25]. Confirmation of successful transfer of the plasmid into the heterologous host was achieved through plasmid isolation and restriction digestions (data not shown). *S. lividan* and *S. lividan* pIBR25-*thnA* were cultivated in R2YE liquid medium for a duration of 5 days at a temperature of 28°C. To extract the cultures, a double volume of ethyl acetate was employed, and the resulting organic supernatant fraction was subsequently concentrated using a rotary evaporator. After concentration, the sample was dissolved in a suitable volume of methanol for subsequent HPLC analysis at a UV wavelength of 330 nm by Thermo HPLC series 1100 with a Thermo-C_18_ column (5 μm, 4.6 mm × 250 mm). The HPLC mobile phase was composed of solvent A (water with 0.1% trifluoroacetic acid [TFA]) and solvent B (acetonitrile) and followed a linear gradient (10% acetonitrile at 0 min, 10 to 90% B for 1–25 min, 90 to 50% B for 25−28 min, and from 50 to 10% B for 28−30 min), at a constant flow rate of 1 ml/min. The HPLC profile showed a distinct peak at a retention time of (*t*_R_) 15.7 min from *S. lividan* pIBR25-*thnA*, but no peak was observed from wild-type or *S. lividan* pIBR25 (Fig. 4A). The identity of THN and flaviolin (an auto-oxidized product of THN) was further supported by LC-ESI/MS analysis ([M+H]^+^ ion at m/z = 193.0489 found, calcd for C_10_H_9_O_4_ 193.0495, and [M+H]^+^ ion at m/z = 207.0284 found, calcd for C_10_H_7_O_5_ 207.0288) (Figs. 4B and 4C).

### RNA Isolation and Real-Time PCR Analysis

**Fig. 5:**
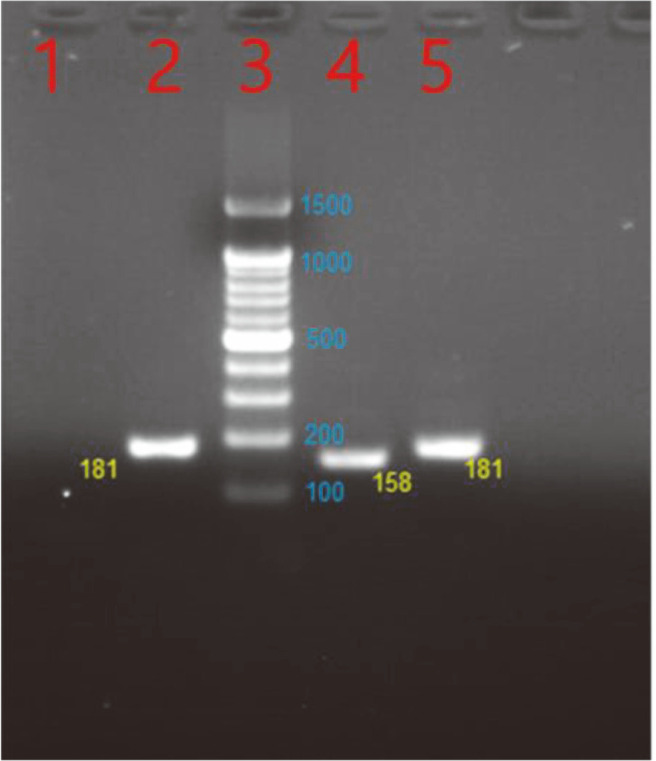
RT-PCR profile of *thnA* and *rpoB* in *S. lividan* TK24; Lane 1: RT-PCR product of *thnA* in *S. lividan*TK24 wild-type, Lane 2: RT-PCR product of *rpoB* as the housekeeping gene in *S. lividan* TK24 wildtype (181 bp), Lane 3: DNA ladder marker, Lane 4: *thnA* (158 bp) in *S. lividan* TK24 pIBR25-ThnA, and Lane 5: *rpoB* (181 bp) in *S. lividan* TK24 pIBR25-ThnA.

For transcriptional analysis of *thnA*, *S. lividan* TK24 and *S. lividan* pIBR25-*thnA* were cultured in 50 ml of HT medium. Samples were taken on the third day, and mRNA was isolated using a standard protocol. Gene-specific primers were used, and *rpoB*, the housekeeping gene in *S. lividan*, was used as a control. The RT-PCR results showed the band of *thnA* (158 bp) in *S. lividan* pIBR25-*thnA*, which did not exist in the wild-type strain, *i.e.*, *S. lividan* TK24, whereas the housekeeping *rpoB* showed a band (181 bp) in both strains (Fig. 5). The amplified sequenced was further confirmed through sequencing after ligation into the pGEM^®^-T Easy vector.

## Conclusion

In this study, we successfully identified and characterized a type III polyketide synthase (ThnA) from Nocardia CS682. The enzyme was found to use five molecules of malonyl-CoA to synthesize 1,3,6,8 tetrahydroxynaphthalene (THN), which was then modified to form the final product 1-(*α*-L-(2-*O*-methyl)-6-deoxymannopyranosyloxy)-3,6,8-trimethoxynaphthalene (IBR-3) with UV-protective properties. To validate its function, we performed the heterologous expression of *thnA* in *S. Lividans* TK24 and confirmed its expression through RT-PCR. In vivo production of flaviolin showed that the enzyme acted as a THN synthase. This characterization provides insight into the biosynthetic pathways of the important compound IBR-3.

## Supplemental Materials

Supplementary data for this paper are available on-line only at http://jmb.or.kr.
