# Supplementary material for: Identification of 1,3,6,8-Tetrahydroxynaphthalene Synthase (ThnA) from Nocardia sp. CS682
Source: J Microbiol Biotechnol. 2023 May 5;33(7):949–54. doi: 10.4014/jmb.2303.03008 (PMC10394336; doi:10.4014/jmb.2303.03008)
Supplement: Supplementary file 1 [file jmb-33-7-949-supple.pdf]

J. Microbiol. Biotechnol.
https://doi.org/10.4014/jmb.2303.03008

Supplementary Figures

Identification of 1,3,6,8-tetrahydroxynaphthalene synthase (ThnA) from *Nocardia* sp. CS682

Purna Bahadur Poudel[1*], Rubin Thapa Magar[1], Adzemye Fovennso Bridget[1], and Jae Kyung Sohng[1,2,*] *

[1]*Institute of Biomolecule Reconstruction (iBR), Department of Life Science and Biochemical Engineering, Sun Moon University, 70 Sun Moon-ro 221, Tangjeong-myeon, Asan-si, Chungnam 31460, Republic of Korea*

[2]*Department of BT-Convergent Pharmaceutical Engineering, Sun Moon University, 70 Sun Moon-ro 221, Tangjeong-myeon, Asan-si, Chungnam 31460, Republic of Korea*

***Corresponding author:** Prof. Jae Kyung Sohng

sohng@sunmoon.ac.kr

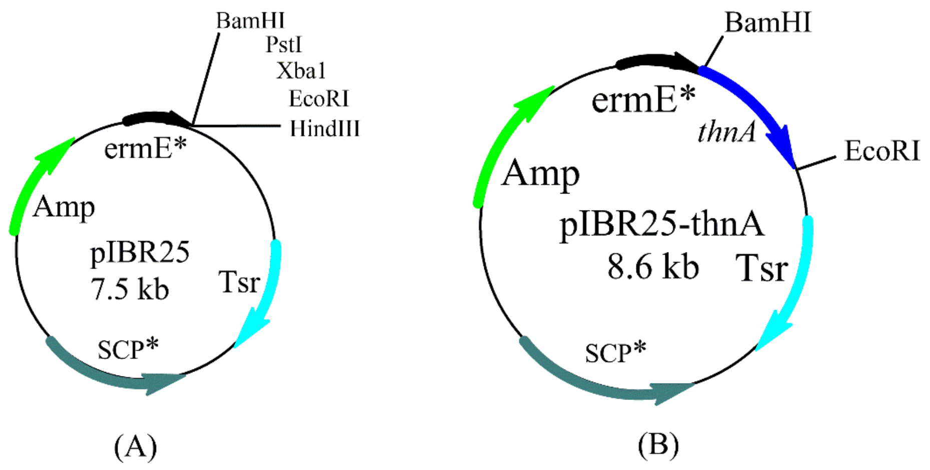

Fig. S1. The vector maps of (A) pIBR25 expression vector and, (B) pIBR25-thnA recombinant plasmids. (supp)

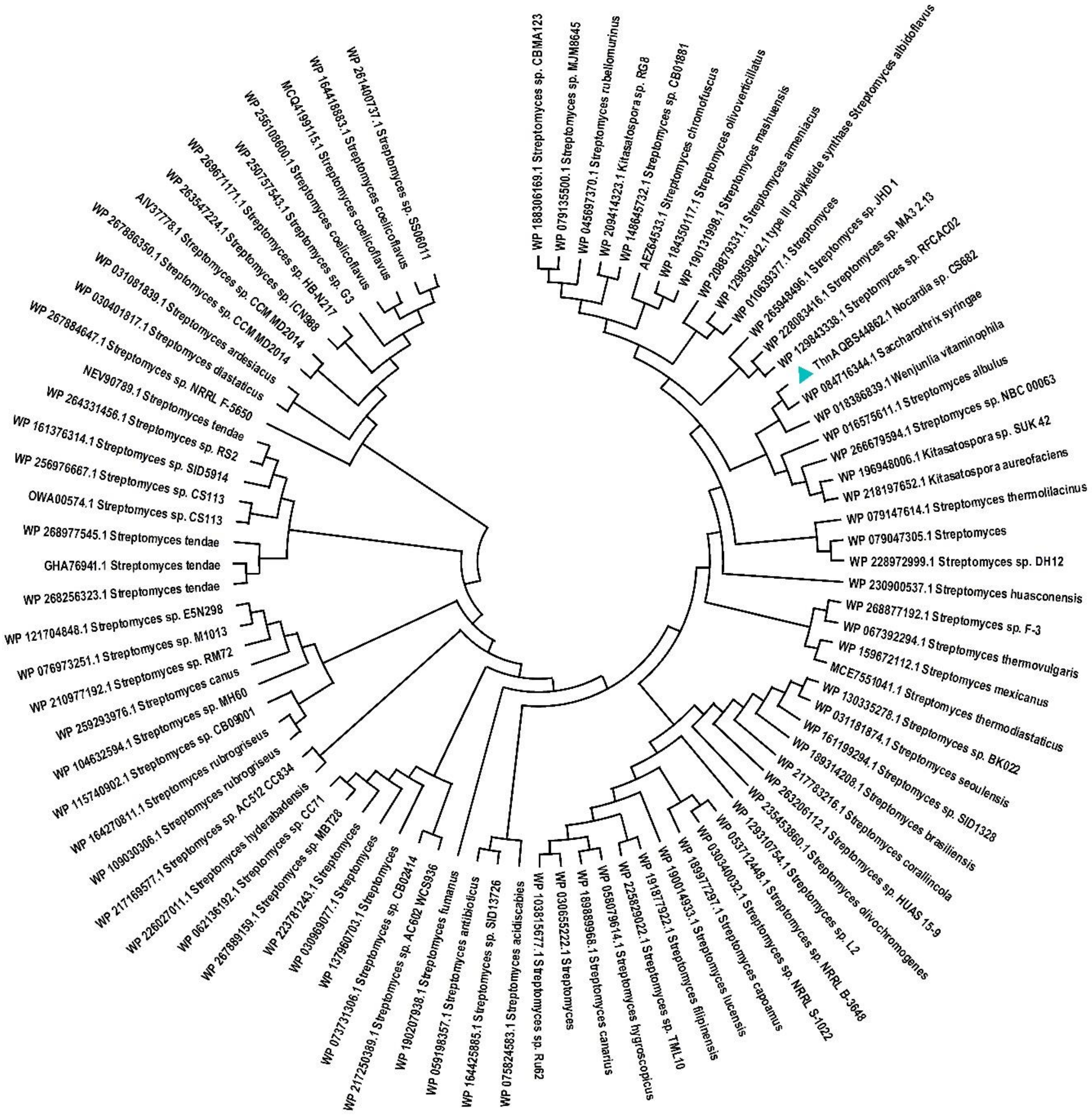

Fig. S2. Evolutionary relationship by type III PKSs family proteins from different sources. The name of the enzyme, strain, and accession number of the amino acid sequence is shown in the tree. Evolutionary analyses were conducted in MEGA X software [1]. The evolutionary history was inferred by using the Maximum Likelihood method and JTT matrix-based model [2]. A total of 88 amino acid sequences were used for the phylogenetic tree analysis. Initial tree(s) for the heuristic search were obtained automatically by applying Neighbor-Join and BioNJ algorithms to a matrix of pairwise distances estimated using the JTT model, and then selecting the topology with superior log likelihood value. The branch length indicates the evolutionary distance between different enzymes. All positions containing gaps and missing data were eliminated. The

significance was tested by bootstrap test (1000 replicates) using MEGA X. *Nocardia* sp.CS682 (ThnA QBS44862.1) is shown in the triangle symbols.

References

1. Jones DT, Taylor WR, Thornton JM. 1992. The rapid generation of mutation data matrices. *Bioinformatics* **8:** 275–282.

2. Kumar S, Stecher G, Li M, Knyaz C, Tamura K. 2018. MEGA X: Molecular evolutionary genetics analysis across computing platforms. *Mol. Biol. Evol.* **35:** 1547–1549.
